# Three-dimensional and two-dimensional echocardiography and biochemical analysis in patients with ST-segment elevation myocardial infarction percutaneously treated: relationship between LV function, remodeling and serum cardiac markers

**DOI:** 10.1186/cc10173

**Published:** 2011-06-22

**Authors:** MLC Vieira, WA Oliveira, AF Cury, A Cordovil, ACT Rodrigues, G Naccarato, CG Mônaco, LPRV Costa, RB Romano, JR Calatróia, TR Afonso, REU Azevedo, GMP Tavares, L Guimarães, EB Lira Filho, MA Perin, CH Fischer, SS Morhy

**Affiliations:** 1 Hospital Israelita Albert Einstein, São Paulo - SP, Brazil

## Introduction

The prognosis of patients with acute myocardial infarction (MI) concerns multiple aspects that demonstrate myocardial aggression (such as serum markers of cardiac damage), and also adaptative mechanisms relative to the acute event (ventricular remodeling).

## Objective

The aim of the study was to assess the relationship of serum markers of cardiac damage and tridimensional echocardiographic (3D Echo) parameters as well as echocardiographic bidimensional (2D Echo) left ventricular ejection fraction (LVEF) in patients with acute ST-elevation MI.

## Methods

A prospective study of 23 patients (17 males, mean age of 57 ± 13 years), with acute ST-elevation MI, primarily percutaneously treated (stent). Serum cardiac markers (CK-MB, troponin I, myoglobin) and serum BNP were compared with echocardiographic parameters (volumes, LVEF, 3D dissynchrony index, 3D sphericity index (3D SPI)). The 3D SPI is defined as: LVEDV/(4/3π(D/2))^3^, where D is left ventricular diastolic diameter on 4CH apical view. 3D SPI was compared with a group of 20 normal volunteers (normal values: 0.29 ± 0.08). The statistical analysis was performed using Pearson's correlation coefficient (*r*), 95% CI, *P *< 0.05, linear regression equation and Bland-Altman test.

## Results

3D SPI ranged from 0.29 to 0.45 (0.35 ± 0.0 8); 3D LVEF ranged from 0.36 to 0.70 (0.50 ± 0.06); 3D EDV ranged from 72 to 159 (100 ± 27) ml; 2D LVEF ranged from 0.40 to 0.71 (0.54 ± 0.08); 2D EDV ranged from 57 to 165 (104 ± 32) ml. Troponin I ranged from 2.3 to 33 (12.9 ± 9) ng/ml; CKMB ranged from 5.7 to 258 (94.4 ± 78) ng/ml; BNP ranged from 25 to 1,058 (264 ± 128) pg/ml. Pearson's correlation coefficient (*r*), relative to 3D LVEF: 1 - BNP: *r *= -0.7427, *P *= 0.4800.

## Conclusion

In this series, stronger correlation was observed relative to serum CK-MB, BNP and 3D Echo LVEF, when compared with 2D Echo LVEF. We did not observe association concerning LV remodeling and cardiac damage assessed by serum cardiac markers.

